# Continuity in a fragmented healthcare system: – organizational and individual determinants

**DOI:** 10.1080/02813432.2026.2690608

**Published:** 2026-06-23

**Authors:** Emil Johansson, Hálfdán Pétursson, Jörgen Månsson, Lina Maria Ellegård, Gustav Kjellsson

**Affiliations:** ^a^ School of Public Health and Community Medicine, University of Gothenburg, Gothenburg, Sweden; ^b^ Department of Family Medicine, Faculty of Medicine, University of Iceland, Reykjavík, Iceland; ^c^ Department of Economics, Lund University, Lund, Sweden; ^d^ Department of Economics and Business Law, Kristianstad University, Kristianstad, Sweden; ^e^ Centre for Health Governance, Department of Economics, University of Gothenburg, Gothenburg, Sweden

**Keywords:** Continuity of care, relational continuity, general practice, primary care, register-based study

## Abstract

**Background:**

Continuity of care is key for high-quality primary care, associated with improved health outcomes, reduced mortality and more efficient use of resources. Achieving relational continuity is challenging in systems with high provider choice and fragmented care-seeking. Sweden exemplifies this tension, combining broad access to primary care and low GP continuity.

**Aim:**

To examine how individual characteristics (age, morbidity, socioeconomic status and migration background) and primary care center (PCC) features (ownership, size, physician turnover, patient mix and location) are associated with relational continuity and to contrast within-PCC continuity with total continuity systemwide.

**Design and setting:**

Retrospective cohort study using linked administrative register data covering all in-person physician contacts in primary care for 1.4 million residents in Region Skåne, Sweden.

**Method:**

Continuity of Care Index (CoCI) was measured using all primary care physician visits over 36-months. Linear regression models estimated associations between individual and PCC characteristics and continuity, adjusting for individual- and PCC-level covariates.

**Results:**

The characteristics most strongly associated with higher continuity were low physician turnover, older age, chronic conditions, smaller PCC size and private ownership. Individuals with higher education, higher income and foreign background had lower total continuity. Differences in continuity were more strongly associated with PCC characteristics than with patient characteristics.

**Conclusion:**

Relational continuity in Swedish primary care is associated primarily with organizational factors, particularly physician turnover and practice size. Fragmented care-seeking among specific groups, especially individuals born outside the Nordic countries, contributes to lower total continuity but does not reflect weaker patient–provider relationships within PCCs.

## Introduction

Continuity of care is a cornerstone of high-quality primary care and a core value of general practice [1]. Sustained continuity has consistently been associated with improved health outcomes, greater patient satisfaction, decreased mortality and more efficient use of resources [2–5]. A well-functioning primary care system is fundamental for achieving public health objectives, such as improving population health, reducing health inequalities and promoting early detection and cost-effective management of chronic conditions [6,7]. Primary care generally serves as the first line of healthcare, managing both acute and chronic conditions not requiring specialized hospital care, and to some extent, providing out-of-hours care [8–10].

International comparison shows substantial variation in the degree of relational continuity, operationalized as the degree to which the patient has a longstanding relationship with a specific caregiver, in the primary care context usually a general practitioner (GP) [11–13]. Countries, such as the Netherlands, Denmark and Norway – where patients are formally or in practice assigned to a named GP in small, GP-led practices – tend to achieve high levels of continuity, supported by strong gatekeeping and systems that promote long-term doctor–patient relationships [14–18]. In contrast, countries like Greece, Ireland and Austria experience low levels of continuity despite the primary care system being based on small GP-led practices [19–21]. The more fragmented and market-driven US system demonstrates low GP continuity, although recent reforms, such as patient-centered medical homes, aim to improve care coordination [22]. Across many countries, there has been a structural shift from small practices to large, multi-professional care organizations [23]. While such changes may enhance accessibility and operational capacity, they may also reduce relational continuity due to higher staff turnover and less consistent provider–patient interactions [23–25].

In fragmented healthcare systems, where patients are free to choose their provider and may seek care from multiple providers, ensuring continuity is inherently challenging [26,27]. In this regard it is useful to consider the concept of longitudinality, defined by Starfield as ‘a phenomenon involving both the availability of a regular source of care and a decision, by the patient, to seek care from that source whenever care is needed’ [28]. The longitudinality concept illustrates that continuity of care is partly determined by the actions of patients. Thus, it is not sufficient for a primary care practice to be organized in a way that promotes high ‘within-practice continuity’, that is, patients consulting the same physician within a practice to a higher degree. ‘Total continuity’ across the primary care system as a whole may still be low if patients consult multiple practices. To gain deeper insight into this potential difference it is important to compare continuity within a specific practice with continuity across the primary care system as a whole.

Swedish primary care illustrates this challenge. It is organized around multi-professional practices, primary care centers (PCCs), where all residents are formally listed. However, patients are generally not assigned to a named GP and may also seek care at other PCCs, including private telemedicine providers and centers outside their home region [29]. This lack of structural support for continuity is likely one of the explanations for why Sweden, despite its broad access to primary care, exhibits relatively low levels of continuity, especially at the GP level [30].

While prior research has identified key individual and organizational factors associated with continuity, such as older age, chronic disease, higher morbidity, rural setting and low physician turnover [31–34], few studies have examined how these factors shape continuity across patient groups and healthcare organizations.

The aim of this study was twofold. First, to examine how patient characteristics (such as age, morbidity, demographics and socioeconomic status) and PCC features (such as turnover rate, size, ownership and urbanicity) are associated with measures of continuity. Second, to compare these associations for within-PCC continuity and total continuity, thereby assessing whether the level of continuity primarily reflects patients’ care-seeking patterns across providers or PCC-level opportunities to offer relational continuity.

## Methods

### Design

This study was a register-based retrospective cohort study using linked administrative data covering all health care contacts in Region Skåne. The design was observational describing differences in continuity between population groups based on individual and PCC characteristics. An individual-level continuity of care measure, Bice–Boxerman Continuity of Care Index (CoCI), was computed using in-person primary care physician contacts over 36 months [35].

### Setting

Region Skåne is the southernmost and the third most populous of Sweden’s 21 administrative regions. The region’s tax-funded health care system serves 1.4 million residents. Primary care is organized as a patient choice system comprising ∼170 PCCs (including around 20 out-of-hours practices), half of which are privately operated. PCCs are multi-professional, primarily staffed by GPs and nurses, with additional access to physiotherapists, occupational therapists and psychosocial expertise. All residents are enrolled at a PCC by their choice. Reimbursement is mainly capitation-based and adjusted for morbidity using Adjusted Clinical Groups (ACG^1^) and for socioeconomic deprivation using Care Need Index (CNI^2^) [36–38].

Residents are free to change PCC at any point in time, and their choice does not restrict them to seek care at a given PCC. Instead, they are free to choose and seek care at any PCC in Sweden. PCCs may decide whether to register patients with a named GP although it should be noted that being registered with a named GP is primarily an administrative arrangement and does not necessarily mean that the patient will see the named GP at each visit [39].

Outside of the patient choice system, there are private providers specializing in telemedicine and a small number of private solo practices both operating on a fee-for-service basis reimbursed according to nationally regulated fee schedules [40–42]. Both types of providers played a limited role during the study period. The number of contacts with private telemedicine providers corresponded to 3.6% of all primary care physician contacts during the study period.^3^

### Data

The main data source was administrative data from Region Skåne’s healthcare utilization database, including information on all healthcare contacts (dates, provider types, practice or clinic identifiers and ICD-10-diagnoses) funded by the region. Continuity was measured using in-person GP visits at PCCs 2017–2019. Morbidity burden was measured using an international risk-classification system, ACG [36]. The individual-level ACG-scores were calculated based on all care contacts in 2016 using a research license from Johns Hopkins University. Indicators for chronic conditions were based on diagnoses from the preceding five years (2012–2016).

The study linked data from several administrative registers by matching records at the PCC level and *via* unique personal identity numbers. Individual-level sociodemographic data (age, sex, education, income, place of residence and country of birth) were obtained from Statistics Sweden (SCB). Geolocation data were used to assign individuals to their closest PCC. Information on PCC characteristics, including size, ownership and physician turnover, was provided by the regional health authority.

### Continuity of care

CoCI is an established measure of continuity of care, assessing the distribution of healthcare encounters across providers. A higher CoCI value indicates greater concentration of visits with the same provider, reflecting better care continuity. CoCI is defined as

$$\text{COCI}=\frac{\sum_{j=1}^{J}(n_{j}^{2})-N}{N(N-1)}.$$

Where *J* denotes the number of unique physicians, $n_{j}$ denotes the number of visits to provider *j* and *N* denotes the total number of physician visits. Thus, CoCI considers both the number of different healthcare providers a patient has visited and the frequency of visits to these providers. The CoCI can range from zero, indicating that all patient visits have occurred with different providers, to one, if all visits have been with the same provider. The more frequently a patient visits the same provider, the higher is the index [35].

CoCI was computed using physician visits solely made within each PCC (within-PCC continuity) and using all physician visits at any PCC (total continuity) over 36 months up until 31 December 2019. The period does not overlap with the COVID-19 pandemic. Within-PCC continuity was computed for each pair of PCC and individual with more than three visits. Thus, individuals may have more than one observation if having visited several PCCs within the time period. To keep the sample of individuals constant between indices, only individuals with three or more visits at a given PCC were included.

### Individual characteristics

Individual characteristics were derived from SCB registers as of 31 December 2017 – two years before the end of the measurement period (2017–2019). Morbidity was captured using two measures, an ACG score computed from 2016 data (categorized into terciles: low, medium and high) and number of chronic diagnoses recorded during 2012–2016 (0, 1–2 and >2). Healthcare use was summarized as the number of visits during 2017–2019, both within the index PCC and across all PCCs. Demographic variables included age (<40, 40–65 and >65) and sex. Socioeconomic status comprised education (primary, secondary and tertiary), country of birth (0 = Nordic, 1 = Western, i.e. Europe (except the Nordic countries)/U.S.A./Canada/Australia, 2 = non-Western, i.e. Africa/Asia/South America) and age-standardized income terciles based on median disposable household income over the preceding five years. Area of residence was proxied by the population size of the nearest PCC catchment area (rural <5000; town 15,000–35,000; urban >35,000). The enrollment indicator captured whether the individual was listed with the same physician at the index PCC at both baseline and at the end of the 36-month period. All categorical variables were coded to include a separate category for missing data. These were included in the analysis as separate categories but are not reported below.

### PCC characteristics

PCC characteristics included ownership (private versus public), location type (rural; town; urban); average deprivation (CNI) and morbidity (ACG) scores; and mean patient list size (2015–2019). Physician turnover was defined using physician contact data (2017–2019) in accordance with previous research [34] and calculated at the PCC-month level as the share of physicians in a given month who remained at the PCC 24 months later. A physician was considered active if they had registered contacts ≥10 days in a month; employment was considered ended if ≥365 days elapsed between contacts. Turnover was then computed as the within-PCC average of the monthly turnover for 2017 and was categorized as low (<25%), medium (25–60%) or high (>60%).

### Statistical analysis

Bivariate and multivariate linear regression models were used to estimate associations between CoCI and a set of individual- and PCC-level characteristics. For each covariate, a baseline unadjusted univariate regression model was first estimated. These unadjusted associations were compared to associations estimated using adjusted multivariate regression models.

For individual-level covariates, two multivariable regression models were estimated: one model including all other individual-level characteristics as controls (denoted as ‘controls’), and one model also including a full set of PCC-specific indicator variables – i.e. PCC-level fixed effects (denoted as ‘controls + fixed effects’). The fixed-effects model exploits variation between patients within the same PCC and adjusts for all observed and unobserved practice-specific characteristics. In practice, this means that the estimated associations reflect differences between patients attending the same PCC, rather than differences between PCCs. Thus, the estimated associations between continuity and individual characteristics should not reflect differences in practice characteristics, such as size, location, practice style or organization of work.

For PCC-level covariates, the baseline model was also compared to two multivariable specifications. The first included individual-level covariates as controls (‘individual controls’), and the second further included other PCC-level characteristics (‘individual + PCC controls’). In all models, standard errors were clustered at the PCC level.

## Results

The study included 375,137 PCC-individual observations with three or more registered primary care visits consisting of 349,661 unique individuals and 172 unique PCCs. The mean total CoCI for all physician visits in primary care was 0.24 in the study population, while the mean individual-level CoCI within each PCC was 0.28. Descriptive statistics for individual and PCC characteristics are presented in Appendix Tables A1 and A2.

**Figure 1.**
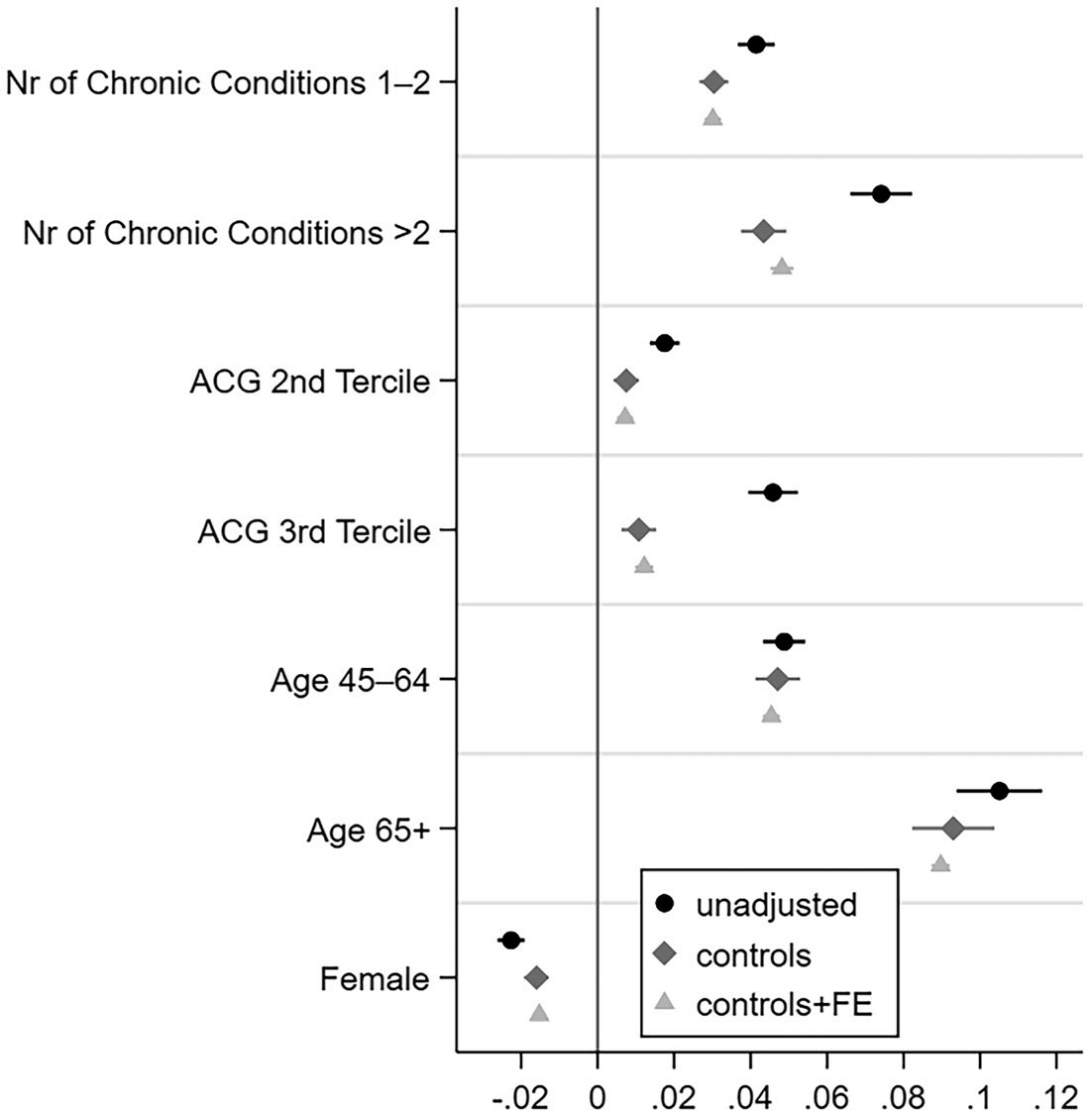
Total continuity of care. Differences in continuity by morbidity (chronic diseases and ACG), age and gender. Unadjusted differences (unadjusted) versus differences adjusted for individual-level characteristics (controls) and adjusted for individual-level characteristics and PCC-level fixed effects i.e. including dummy variables indicating the individual PCC (controls+FE). Dots represent coefficient estimates and horizontal lines indicate 95% confidence intervals.

Age, chronic conditions and greater morbidity (ACG) were associated with higher CoCI, even after adjustments for other individual characteristics and PCC-level fixed effects (Figure 1). Having chronic conditions showed a stronger and more robust association with CoCI than individual ACG score, remaining stable across all model specifications. Gender differences were also observed, with female patients having lower continuity than male patients.

**Figure 2.**
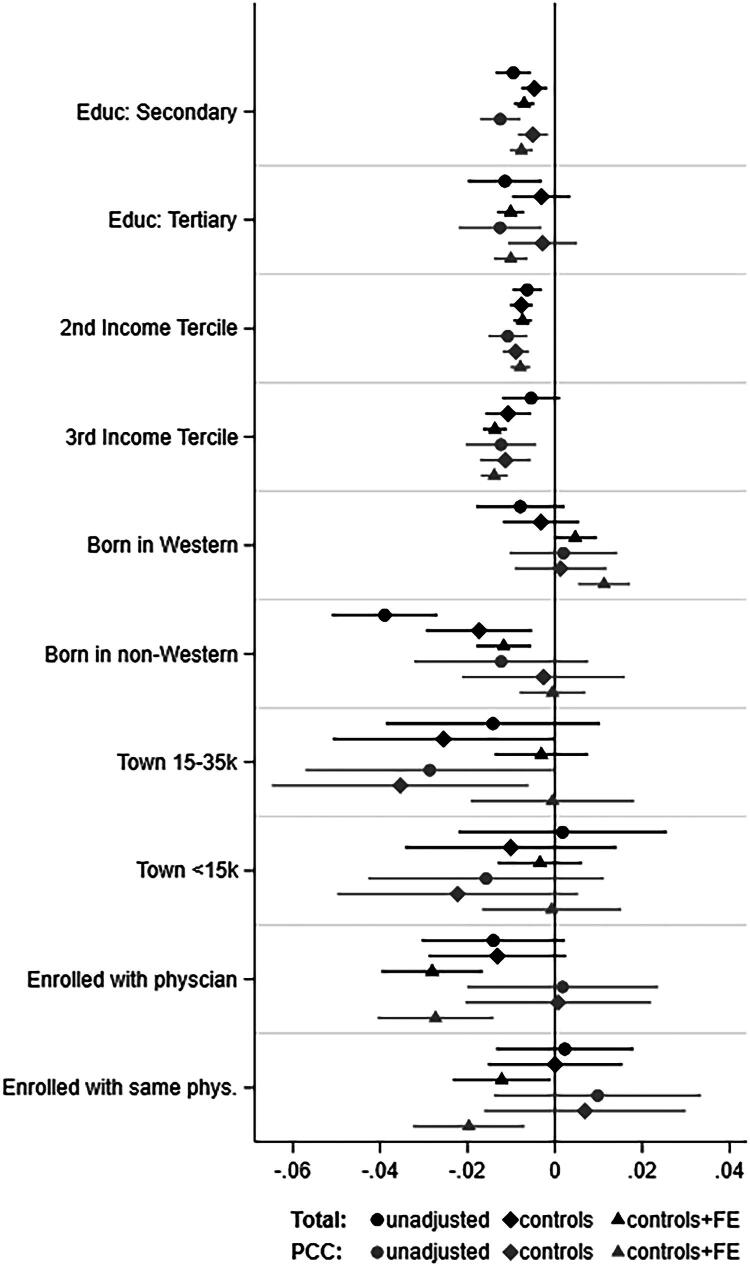
Continuity of care; total versus within-PCC. Differences by education (terciles), income (tercile), birthplace (Nordci vs. Western vs. Non-Western) and place of residence (Urban vs. Town 15-35k vs Town <15k). Unadjusted differences (unadjusted) versus differences adjusted for individual-level characteristics (controls) and adjusted for individual-level characteristics and PCC-level fixed effects (i.e. including dummy variables indicating the individual PCC). Dots represent coefficient estimates and horizontal lines indicate 95% confidence intervals.

Individuals with higher educational attainment and higher income tended to have lower CoCI. These patterns were more pronounced when measuring within-PCC continuity (Figure 2).

The difference in continuity between Nordic-born individuals and individuals born in other countries varied by regression model and by whether continuity was measured in total or within-PCC. Individuals born in Western countries had significantly higher continuity than those born in the Nordic countries when comparing individuals within the same PCC (i.e. +0.011 in the controls + fixed-effects model). This pattern was more evident for within-PCC continuity than for total continuity. Those born in non-Western countries were observed to have lower total continuity compared to Nordic-born, although it was less pronounced after adjustment. There was no such difference when measuring within-PCC continuity (Figure 2).

**Figure 3.**
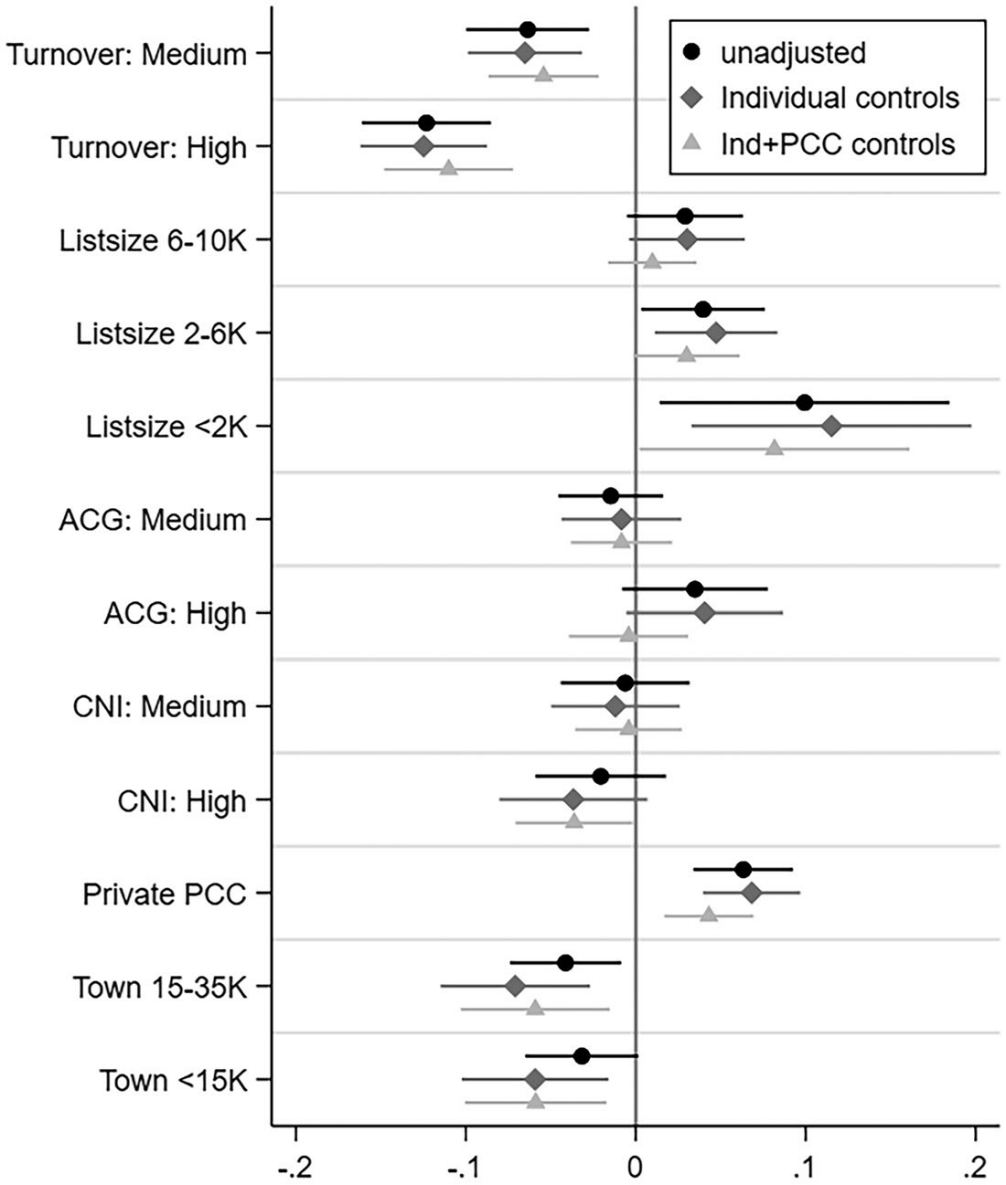
Continuity of care by PCC-level determinants. Differences by turnover (low vs. medium vs. high), list size (<2K vs. 2-6K vs. 6-10K vs. >10K), ownership (Private vs. Public), location (Urban vs. Town 15-35k vs Town <15k) and patient mix based on morbidity (ACG: low vs. medium v.s high) and deprivation (CNI: low vs. medium vs. high). Unadjusted differences (unadjusted) versus differences adjusted for individual-level characteristics (individual controls) and adjusted for individual-level characteristics and PCC-level characteristics (Ind + PCC controls). Dots represent coefficient estimates and horizontal lines indicate 95% confidence intervals.

Living in an urban area was associated with higher total CoCI compared to residing in a medium-sized town, although the difference between urban and rural areas was not statistically significant (Figure 2). When comparing individuals within the same PCC using PCC-fixed effects, continuity did not differ between individuals depending on place of residence. In contrast, when comparing mean-level continuity between primary care units (Figure 3), continuity was higher in the most urban category. This difference was more pronounced than the urban–rural variation observed at the individual level.

Individuals enrolled with a physician – whether defined as being listed with a physician or with the same physician – did not exhibit higher continuity of care. In the fully adjusted model, comparing individuals within the same practice, continuity was in fact lower among these individuals.

Exploring how PCC characteristics relate to CoCI (computed within the PCC) revealed some differences. The largest difference was between the PCCs in the highest and lowest quartile of physician turnover: 0.11 in the fully adjusted model. In relative terms, CoCI was 32% larger in the highest compared to the lowest quartile (Figure 3). Individuals at PCCs with more registered patients had lower CoCI than those at PCCs with fewer ones; the (adjusted) difference in means between the smallest PCCs (<2000 patients) and the reference group of the largest units (>10,000) was 0.080 corresponding to a 31% difference relative to the mean CoCI in the reference group.

Furthermore, individuals at private PCCs had higher continuity than those at publicly operated ones, even when adjusting for individual and PCC-level characteristics. Regarding differences related to patient mix, continuity was lower among patients at PCCs with a higher deprivation index (CNI), though the difference was only statistically significant in the fully adjusted model. The difference between PCCs with high and low ACG score (capturing the average morbidity of enrolled patients) was not statistically significant in any model, and it was very close to zero after adding PCC-level controls.

## Discussion

This study on organizational-level and individual-level determinants of relational continuity in primary care identified low physician turnover rate as the factor most strongly associated with higher CoCI, followed by older age, smaller list size, having chronic medical conditions and private ownership of the PCC.

The distinction between total and within-PCC continuity offers important new insights into how healthcare structure shapes patient–physician relationship. The difference between these two measures was most evident among individuals with foreign background, in particular individuals from countries with a larger cultural distance to Sweden (i.e. non-Western countries). This group had lower total continuity, compared to the reference group of Nordic-born individuals, but there was no difference when continuity was measured within the PCC (adjusting for other characteristics). These patterns indicate that disparities in continuity between individuals with different countries of birth are mainly attributable to differences in care-seeking behavior and less so to differences in continuity within the practice. Previous studies have documented that foreign-born have a greater tendency to seek care from multiple providers as well as having lower continuity of care [43–46]. The novelty in this study is demonstrating the interrelation of these that these patterns: lower continuity among the non-Western individuals is largely attributable to more fragmented care-seeking behavior. While individuals born in Western countries had higher continuity, both within the PCC and across the whole primary care system, compared to Nordic-born ones, the difference was smaller for total continuity. Thus, fragmented care-seeking behavior reduced the continuity for this group of foreign-born as well.

In contrast to previous research from the study region [30], which relied on less granular geographical data, this analysis shows that continuity differs depending on where both the individual and the PCC are located. Continuity was higher in cities, particularly in comparison with PCCs located in smaller towns. However, the difference was slightly smaller when examining total rather than within-PCC continuity, suggesting that the greater opportunities to seek care from multiple providers in urban areas reduce total continuity.

The results further indicate that, in accordance with previous studies, continuity was highest among patients with the greatest care needs, suggesting that even in a low-continuity context, those who likely benefit the most are prioritized [11,21,31,32]. In line with previous studies individuals with lower socioeconomic status had higher continuity, although these differences were small [44,47]. As the pattern was consistent for both total and within-PCC continuity, the lower continuity among the more advantaged groups was unlikely a result of more fragmented care-seeking behavior. Instead, the inverse socioeconomic gradient may reflect higher health literacy and a greater propensity to seek care from specialist providers among individuals with higher socioeconomic status. These individuals may also be better equipped to navigate the healthcare system and, consequently, less reliant on their registered PCC as the primary point of contact [48–50]. In contrast to this inverse socioeconomic gradient, the analysis of PCC characteristics suggested that individuals at PCCs with a more deprived patient mix had lower continuity.

Overall, results indicate that continuity to a large degree depends on the type of PCC the individual attends. Except for differences related to patients’ healthcare needs, the differences across PCC characteristics were more pronounced than differences between patient subgroups. Smaller practices have been shown to have higher continuity [25,51,52] whereas the sparse literature on the effects on continuity by management form is less clear [53].

Staffing stability plays a key role in explaining differences in continuity across units [54]. Centers with high physician turnover showed substantially lower continuity, even after accounting for differences in size and ownership. High staff turnover has a central role as it may undermine continuity both directly, through the disruption of patient–physician relationships, and indirectly, by impeding organizational initiatives to improve continuity, as shown in previous research [55,56].

From a policy perspective, it is noteworthy that individuals who were enrolled with a physician (or even the same physician throughout the study period) and not only with a PCC, did not have higher continuity of care. In the fully adjusted model, these patients even had lower within-PCC continuity. The absence of a positive effect illustrates that formal structures alone are insufficient. This is in line with findings from studies in both England and Sweden [39,57] and a review article on several high-income countries with universal health coverage [58]. The latter article argues that the effectiveness of registration schemes depends partly on the availability of GPs within the health system. In systems where there are fewer GPs per capita (as in Sweden) the effects of patient registration in achieving better integration and coordination are limited [58].

## Strengths and limitations

A key strength of this study is the use of comprehensive register data covering all primary care visits in Sweden’s third-largest region, providing a solid empirical basis for generalizability to other Swedish regions and, to some extent, other healthcare systems. The data link complete utilization records with individual-level information on morbidity and sociodemographic characteristics for the region’s entire population, and include PCC-level features, such as physician turnover, patient list size and ownership. Furthermore, the dual-measure approach – distinguishing between continuity within a given practice (within-PCC) and continuity across all providers (total) – helps clarify the levels at which fragmentation occur and offers insights relevant to fragmented healthcare systems with high provider choice, a context increasingly common internationally as patient choice reforms are introduced.

However, several limitations should be acknowledged. First, the generalizability of our findings is partly limited by the specific institutional context of Swedish primary care. While associations between continuity and PCC-level factors, such as physician turnover, list size and patient morbidity, are likely to generalize across healthcare systems, the magnitude of the gap between total and within-PCC continuity is likely specific to systems with weak registration rules and high provider choice.

Second, applying CoCI to measure the concentration of visits does not fully capture the length or depth of the relationship [59]. Although CoCI has been shown to correlate strongly with alternative quantitative measures of continuity in other contexts [60,61], it does not account for informational or management continuity [62]. Third, the study fails to capture how access to close substitutes for in-person physician visits at PCCs affects continuity of care. For patient groups who maintain continuity with other healthcare professionals or with physicians outside the PCC, the study may underestimate continuity. For patient groups with fragmented care-seeking patterns, remote telemedicine visits may further decrease continuity. Notably, telemedicine played a limited role during the study period, accounting for 3.6% of all primary care physician contacts. The implications for continuity are, however, likely more pronounced after the expansion of these services following the COVID-19 pandemic. Fourth, the study design does not permit causal inference. The study describes differences in continuity between patient groups and PCCs, and the observed associations should not be interpreted as causal effects. Several sources of confounding may influence the results. For instance, patients with stronger preferences for continuity may self-select into PCCs with low physician turnover, and PCCs serving patients with more fragmented care-seeking will mechanically record lower continuity regardless of their organizational capacity. Furthermore, there may be residual confounding from unobserved differences in health status. For example, individuals with higher socioeconomic status may have better health than captured by available morbidity measures, which could partly explain lower continuity of care.

More broadly, the observational design limits the extent to which the associations between PCC characteristics, such as private ownership and list size, and continuity can be attributed to specific organizational mechanisms. Exploring the extent to which these patterns reflect organizational factors that can be adopted by other practices is an important topic for future research.

## Implications for policy and practice

Physician turnover was the factor most strongly associated with continuity, underscoring workforce retention as a central policy priority. Although evidence on effective strategies in the Swedish context remains limited, international research indicates that addressing workload pressures, supporting professional autonomy and fostering stable team-based work are essential for retaining physicians within the same practice [34,63,64].

The results further highlight that the Swedish institutional setting, which allows patients to seek care at any PCC, may reduce the system’s ability to promote relational continuity, particularly for groups whose care-seeking patterns tend to be fragmented across multiple providers [29,30]. The differences between total and within-PCC continuity for foreign-born patients indicate that patient choice without adequate structural navigation support risks widening inequalities in relational continuity [44].

PCCs serving more socioeconomically deprived patient populations exhibited lower continuity of care [56]. Because the results do not show that more deprived patients receive lower continuity within the same PCC, the observed disparities are likely attributable to structural differences between PCCs located in more or less deprived areas. One such difference is the higher rate of GP turnover in PCCs in deprived areas [34]. Strategies aimed solely at stabilizing staffing may not be sufficient, as the association between deprivation and continuity persisted even after adjusting for turnover rates. PCCs serving more deprived populations may need additional organizational changes or stronger structural support – through resource allocation, targeted recruitment or adjusted payment models – to ensure that continuity of care is not contingent on the patient’s background.

## Supplementary Material

Supplementary Material.docx

## Data Availability

The dataset used in the analysis contains sensitive personal information and cannot be publicly shared, but it can be made accessible to qualified researchers for replication purposes under appropriate confidentiality agreements.

## Footnotes

1 The ACG system summarizes a patient’s morbidity by grouping diagnoses over 18 months. Individual risk adjustment scores are aggregated to describe population morbidity and predicted care need.

2 CNI is a socioeconomic index reflecting the expected workload based on patients’ social and demographic characteristics, such as age, education, employment, family structure and migration background.

3 Authors’ own calculations using data obtained from Region Skåne and the Swedish Association of Local Authorities and Regions.

## Generative artificial intelligence

During the preparation of this manuscript, the authors used Claude (Anthropic) and ChatGPT-4 (OpenAI) for language editing and refinement of text. The authors take full responsibility for the integrity and accuracy of the published work.

## Credit statement

EJ: conceptualization; formal analysis; funding acquisition; investigation; methodology; validation; visualization; writing – original draft; writing – review & editing.

JM: conceptualization; funding acquisition; investigation; methodology; validation; visualization; writing – original draft; writing – review & editing.

HP: conceptualization; funding acquisition; investigation; methodology; validation; visualization; writing – original draft; writing – review & editing.

LME: data curation; funding acquisition; investigation; methodology; writing – review & editing.

GK: conceptualization; data curation; formal analysis; funding acquisition; investigation; methodology; software; resources; validation; visualization; writing – original draft; writing – review & editing.
