# Supplementary material for: Continuity in a fragmented healthcare system: – organizational and individual determinants
Source: Scand J Prim Health Care. 2026 Jun 23;44(1):2690608. doi: 10.1080/02813432.2026.2690608 (PMC13292299; doi:10.1080/02813432.2026.2690608)
Supplement: Supplementary Material.docx [file IPRI_A_2690608_SM8213.docx]

Appendix Table A1: Descriptive statistics individual level characteristics

|  | Proportion | # observations |
| --- | --- | --- |
| No Chronic Condition | 0.646 | 225906 |
| Number of Chronic Conditions 1-2 | 0.232 | 81093 |
| Number of Chronic Conditions >2 | 0.122 | 42662 |
| ACG score 1st Tercile | 0.316 | 110361 |
| ACG score 2nd Tercile | 0.335 | 117170 |
| ACG score 3rd Tercile | 0.346 | 121023 |
| ACG missing | 0.003 | 1107 |
| Female | 0.586 | 204877 |
| Age <45 | 0.165 | 57825 |
| Age 45-64 | 0.353 | 123575 |
| Age 65+ | 0.481 | 168261 |
| Number of visits <5 | 0.343 | 120030 |
| Number of visits 5-9 | 0.455 | 159084 |
| Number of visits >9 | 0.202 | 70547 |
| Education, less than secondary | 0.274 | 95843 |
| Educ, any secondary | 0.423 | 147766 |
| Educ, any tertiary | 0.303 | 106052 |
| Income 1st Tercile | 0.329 | 115001 |
| Income 2nd Tercile | 0.327 | 114472 |
| Income 3rd Tercile | 0.323 | 112978 |
| Income missing | 0.021 | 7210 |
| Born in Nordic countries | 0.818 | 285884 |
| Born in Western | 0.099 | 34617 |
| Born in non-Western | 0.083 | 29160 |
| City >35K | 0.433 | 151255 |
| Town 15-35K | 0.198 | 69237 |
| Town <15K | 0.369 | 129169 |
| Enrolled with a physician | 0.532 | 186120 |
| Enrolled with the same physician | 0.262 | 91585 |

**Note:** Individual characteristics measured as of 31 December 2017. Morbidity was captured using ACG scores (categorized into terciles based on 2016 data) and number of chronic diagnoses recorded during 2012–2016. Education was categorized as less than secondary, secondary, or tertiary. Income terciles are age-standardized based on median disposable household income over the preceding five years. Country of birth: Western = Europe (excluding the Nordic countries), USA, Canada, and Australia; non-Western = Africa, Asia, and South America. Area of residence is categorized by the location of the nearest PCC: city (>35,000 inhabitants), town (15,000–35,000), and small town (<15,000). Enrolment is captured by two indicators: whether the individual was listed with any named physician at the index PCC, and whether they were listed with the same named physician at both the start and end of the 36-month period. Missing values are reported as separate categories where applicable and were included as such in the regression analyses.

Appendix Table A2: Descriptive statistics PCC level characteristics

|  | Proportion | # observations |
| --- | --- | --- |
| Low physician turnover | 0.203 | 35 |
| Medium physician turnover | 0.471 | 81 |
| High physician turnover | 0.203 | 35 |
| Turnover missing | 0.122 | 21 |
| List size >10K | 0.291 | 50 |
| List size 6K -10K | 0.343 | 59 |
| List size 2K -6K | 0.256 | 44 |
| List size <2K | 0.035 | 6 |
| List size missing | 0.076 | 13 |
| Public PCC | 0.494 | 85 |
| Private PCC | 0.430 | 74 |
| Out-of-hours unit | 0.076 | 13 |
| CNI: low | 0.238 | 41 |
| CNI: medium | 0.465 | 80 |
| CNI: high | 0.221 | 38 |
| CNI: missing | 0.076 | 13 |
| ACG: low | 0.221 | 38 |
| ACG: medium | 0.459 | 79 |
| ACG: high | 0.244 | 42 |
| ACG: missing | 0.076 | 13 |
| City >35K | 0.424 | 73 |
| Town 15-35K | 0.157 | 27 |
| Town <15K | 0.343 | 59 |
| PCC rural missing | 0.076 | 13 |

**Note:** PCC characteristics derived from regional health authority data. Physician turnover was calculated as the share of physicians active in a given month who remained at the PCC 24 months later, averaged over 2017 and categorized as low (<25%), medium (25–60%), or high (>60%). List size is the mean number of enrolled patients over 2015–2019. CNI (Care Need Index) reflects the expected workload based on patients' social and demographic characteristics. ACG reflects the average morbidity of enrolled patients. Area of residence is categorized by the location of the nearest PCC: city (>35,000 inhabitants), town (15,000–35,000), and small town (<15,000). Missing values are reported as separate categories where applicable and were included as such in the regression analyses.
